# Impact of Intravitreal Dexamethasone Implant on Vessel Diameters in Patients with Retinal Vein Occlusion

**DOI:** 10.1155/2019/3982428

**Published:** 2019-04-02

**Authors:** Busra Yilmaz Tugan, Levent Karabas, Berna Ozkan

**Affiliations:** ^1^Izmit Seka State Hospital, Department of Ophthalmology, Kocaeli, Turkey; ^2^Kocaeli University, Department of Ophthalmology, Kocaeli, Turkey; ^3^Acıbadem Mehmet Ali Aydınlar University, Department of Ophthalmology, Istanbul, Turkey

## Abstract

**Purpose:**

To investigate the vasomotor responses and diameter of retinal vessels in patients with macular edema secondary to retinal vein occlusion (RVO) who were treated with intravitreal dexamethasone implant.

**Methods:**

We enrolled 17 eyes of 17 patients with macular edema secondary to RVO. All patients were evaluated through optical coherence tomography and dynamic and static retinal vessel analysis, using the Dynamic Vessel Analyzer (Imedos, Jena, Germany) before administration (baseline) and 1 week, 1 month, and 2 months after administration of intravitreal dexamethasone. Measurements of patients were compared to those of 17 eyes of age- and sex-matched control subjects.

**Results:**

In static analysis, arteriovenous ratio (AVR) in control subjects was 0.86 (0.80–0.88). In RVO patients, baseline AVR was 0.71 (0.54–0.84) and significantly lower than that in control subjects (*p*=0.016). Baseline AVR in the RVO group was significantly lower than AVR at month 1 and month 2 (*p*=0.001 and *p* < 0.001, respectively). CRVE in healthy control subjects was 183.59 ± 21.79 measurement units (MU) which was significantly different from CRVE of RVO eyes at baseline (207.00 ± 26.35 MU) (*p*=0.008). Static analysis showed a significant decrease of central retinal vein equivalent (CRVE) from baseline to 1 week, 1 month, and 2 months (*p* < 0.001, *p* < 0.001, and *p* < 0.001, respectively). CRAE in the control group was 176.24 ± 22.45 MU. CRAE in the RVO group was significantly lower at baseline, week 1, month 1, and month 2 compared to that in the control group (*p*=0.008, *p*=0.003, *p*=0.013, and *p*=0.011, respectively). Dynamic analysis showed that maximum venous and arterial dilations did not statistically differ from baseline to 1 week, 1 month, or 2 months.

**Conclusion:**

Using the Dynamic Vessel Analyzer, we found that retinal veins in patients with RVO were significantly larger compared to those in the control group, and intravitreal dexamethasone treatment reduced the diameters of these veins.

## 1. Introduction

Retinal vein occlusion (RVO) is the second most common cause of visual loss in retinal vascular diseases [1]. RVO is a vascular disorder characterized by dilation and engorgement of the retinal veins. Intraretinal hemorrhage, macular edema, cotton wool spots, exudates, and retinal ischemia can be seen [2].

Arteriosclerosis and hypertension may be the cause of central retinal vein occlusion (CRVO) by leading to increased rigidity of central retinal artery which forms an adventitial sheath in common with the adjacent central vein. This relationship facilitates compression of same vein [3, 4]. At lamina cribrosa level, central venous lumen can be compressed by the aid of predisposing factors, and turbulent flow may lead to vein obstruction [3, 4]. In branch retinal vein occlusion (BRVO) pathogenesis, crossing artery over a vein leading turbulent blood flow and impairment of endothelium with thrombus formation has a key role [5–7].

Cystoid macular edema (CME) and macular ischemia are the most important reasons of visual loss associated with either CRVO or BRVO. Inflammation has an important role in pathogenesis of RVO. Induction of inflammatory mediators upregulation (such as vascular endothelial growth factor, tumor necrosis factor-a, prostaglandins and leukotrienes) and chronic, low-grade inflammation of retinal microvasculature by damaged endothelium may facilitate macular edema (ME) [8].

Corticosteroids act by inhibiting the metabolic pathway of the vascular endothelial growth factor (VEGF), expression of the VEGF gene, and intervene in inflammatory processes causing vasodilation, exudation, and edema in ME related to uveitis, diabetic retinopathy, or RVO [9]. Steroids are able to stabilize endothelial cell tight junctions, leading to reduce vascular permeability and cause inhibition of proinflammatory mediators such as prostaglandins, leukotrienes, and several cytokines [10, 11]. Also change within inflammatory cascade of cytokines by treatment with intravitreal corticosteroids exhibits vasomotor effects and may therefore influence vessel diameters and dilation rates [12]. Intravitreal injection of 0.7 mg dexamethasone implant (Ozurdex; Allergan, Inc., Irvine, CA, USA) was introduced as a therapeutic option for treating ME in RVO patients. DEX intravitreal implant provides continuous release of DEX for up to 6 months [13]. Functional and morphologic outcome of DEX implant in RVO has been analyzed by some studies [14, 15].

Dynamic Vessel Analyzer (DVA; Imedos Systems, Ltd, Jena, Germany) is a device which measures vascular dilation rates and vascular diameters and also calculates arteriovenous ratios. The device works by measuring different parameters both at static and dynamic conditions.

In the present study, we performed a vascular function analysis in patients with ME secondary to BRVO or CRVO treated with DEX implant and in a healthy control group using the Dynamic Vessel Analyzer to understand DEX effects on vascular diameters and dilation rates in patients with RVO.

## 2. Methods

### 2.1. Study Participants and Protocol

We obtained an informed consent from all patients for performing this observational study in agreement with the Declaration of Helsinki for research involving human subjects. The Ethics Committee of the Kocaeli University approved the study. Patients presenting with decreased vision because of RVO-related macular edema were enrolled in the study. Criteria for inclusion were (1) ME (central macular thickness (CMT)) > 300 mm, as measured by spectral domain optical coherence tomography (SD-OCT, Spectralis; Heidelberg Engineering, Heidelberg, Germany) secondary to RVO, (2) best-corrected visual acuity (BCVA) between 5 and 50 Early Treatment Diabetic Retinopathy Study (ETDRS) letters in the study eye, and (3) age above 18 years. The exclusion criteria were (1) any ocular surgery in the past 6 months, (2) previous intravitreal injection of anti-VEGF in the last 3 months, (3) intravitreal corticosteroid injection in the last 6 months, (4) diabetic retinopathy, (5) ocular inflammation history, (6) significant media opacities, (7) single-eyed patients, and (8) patients who could not follow consecutive examinations. Also, age- and sex-matched healthy control subjects were recruited in the study.

All subjects (RVO patients and control group) underwent ophthalmic evaluation, including assessment of distance BCVA using ETDRS charts, tonometry, slit-lamp biomicroscopy and indirect fundus ophthalmoscopy, fluorescein angiography, SD-OCT with automated CMT measurements, customized high-resolution enhanced depth imaging (EDI) SD-OCT scans, and dynamic and static analyses with the DVA. The same day, all patients received dexamethasone implant. Follow-up examinations after treatment were performed at 1 week, 1 month, and 2 months and included distance BCVA, tonometry, slit lamp biomicroscopy, indirect fundus ophthalmoscopy, SD-OCT, EDI SD-OCT, and DVA examination. During the study period, control subjects also underwent ophthalmic evaluation, which included SD-OCT and EDI-SD OCT scans and DVA examination.

### 2.2. Dynamic Vessel Analysis

The DVA is a technique which enables evaluation of retinal blood vessels and accurately measures the response of retinal vessels to diffuse luminance flicker light easily and noninvasively. Flicker light stimulation facilitates retinal regulation of blood flow in response to neural activity. Flicker stimulation induction of neural activity by releasing vasodilating factors like nitric oxide which is released from endothelial and neural cells causes retinal arterial and venous dilation.

**Figure 1:**
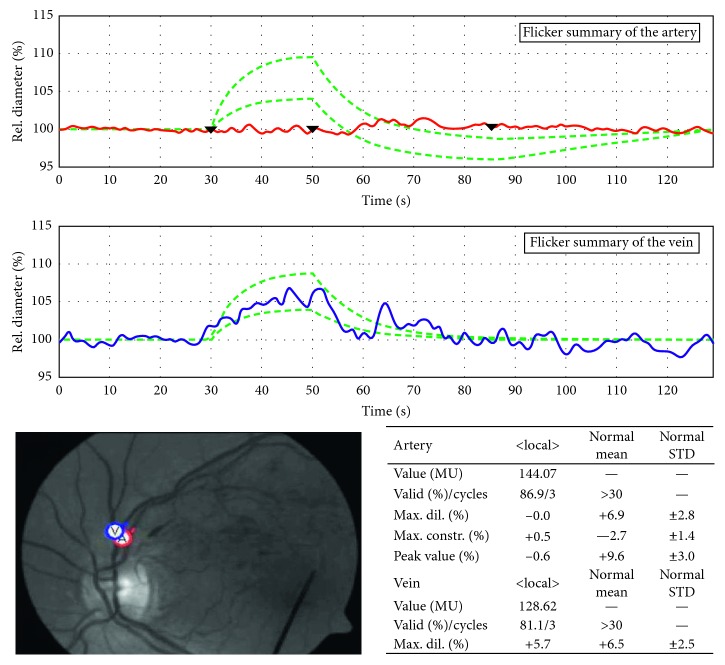
Dynamic vessel analysis of patient #3 with macular edema secondary to BRVO. In patients with RVO, arterial and venous segments were chosen and marked with a probe (red for the artery and blue for the vein) (bottom left) to evaluate the arterial (first row) and venous (second row) flicker response.

Measurements were performed before the injection (baseline) and after 1 week, 1 month, and 2 months. Pupil dilation was obtained with topical tropicamide. Patients informed to stay focused on the tip of a fixation bar during the test while the fundus was examined under green light with an average luminance of 130 cd/m^2^ (ILT1700 Research Radiometer; International Light Technologies, Peabody, Massachusetts, USA). A great contrast between retinal blood vessels and the adjacent tissue using a green light illumination (530–600 nm) is created by the DVA, while in contrast, most surrounding tissue reflects light in this range [16]. The DVA contains software to track the eye movements, so once a focus image of the fundus was obtained, a fixation target was located in a single zone (e.g., a vessel branch) over the entire 30-degree visual field. The same method by Corvi et al. [17, 18] was used to perform the dynamic analysis in patients with CRVO and in healthy controls. A superior or inferior temporal venous and arterial segment located between one half and 2 disc diameters from the optic disc margin and at least 1 vessel diameter from any bifurcation or close vessel was chosen and marked with a probe (blue for the vein and red for the artery) (Figure 1). The same procedure was performed in patients with BRVO; in this case; the venous branch occluded with corresponding arterial segment located between one half to two disc diameters from the optic disc margin and at least one vessel diameter from any bifurcation or close vessel was chosen. The examination duration was 350 s, which included 3 cycles of flicker/nonflicker light. Flicker with an optoelectronic shutter that interrupts the light source with a bright-to-dark ratio of 25 : 1 at a frequency of 12.5 Hz is created by the DVA, to maximize vasodilation and blood flow during flicker [19–21]. Selected vessel diameters were first recorded for 50 s, then a flicker stimulation was applied for 20 s, followed by a nonflicker period for 80 s; the sequence was repeated 3 times [21, 22]. Vessel diameters were calculated and expressed in measurement units (MU); vessel dilation was measured by calculating the percentage increase in vessel diameter relative to baseline after 20 s of flicker stimulation and averaging the 3 measurement cycles.

The dynamic analysis is a real-time examination in which the operator can follow the analysis on a monitor video. He can realize if patients have a proper fixation on the tip of a bar. Also, the arterial and venous tracks appear second by second on the monitor video that allow to show if patients have a proper fixation. Moreover, with the report the DVA provides a validity percentage score. In case of an improper fixation for a sufficient time, the DVA does not provide the result.

### 2.3. Static Vessel Analysis

**Figure 2:**
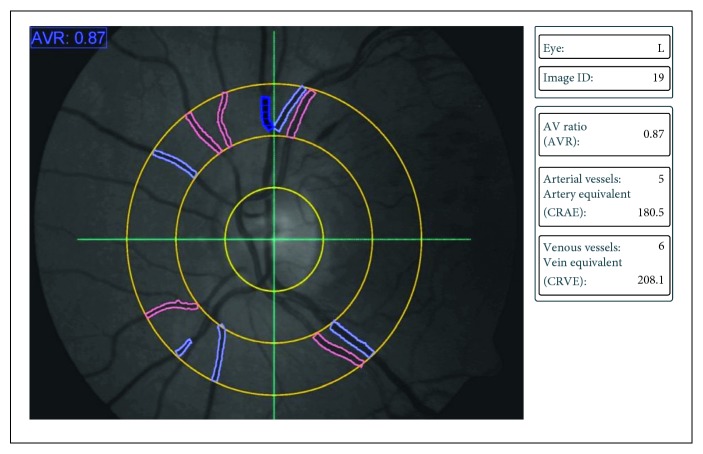
Static vessel analysis of patient #3 with macular edema secondary to branch retinal vein occlusion (BRVO). After fundus photograph was taken, arterial and venous vessels were manually selected and central retinal artery equivalent, central retinal vein equivalent, and arteriovenous ratio were calculated.

By using the FF450 retinal camera (Zeiss AG, Jena, Germany) contained in the DVA system, a 50-degree fundus photograph was taken. These photographs were analyzed by VISUALIS and VesselMap Software (Imedos Systems, Ltd, Jena, Germany) (Figure 2). Also as in Corvi et al. [17, 18] studies, using an optic disc-centered image, the papilla is marked and the software creates an area of one-half to one disc diameter from its center to measure all vessels. Arterial and venous vessels are selected (Figure 2). In all subjects and also BRVO and CRVO groups, we calculated the central retinal artery equivalent (CRAE), which relates to the diameter of the central retinal artery; the central retinal vein equivalent (CRVE), which relates to the diameter of the central retinal vein; and the arteriovenous ratio (AVR), which represents the CRAE/CRVE ratio at baseline and 1 week, 1 month, and 2 months after dexamethasone injection. These measurements were compared with control group and also measurements at 1 week, 1 month, and 2 months after injection were compared with baseline. In all patients with RVO and also BRVO and CRVO subgroups, we computed arteriovenous ratio, CRAE, and CRVE of each single (affected/occluded and unaffected/nonoccluded) quadrant (superior nasal, inferior nasal, superior temporal, and inferior temporal). Measurements at 1 week, 1 month, and 2 months after injection were compared with those of control group and baseline. Also arterial and venous dilation rates, artery and venous diameters of all RVO patients, and also CRVO and BRVO subgroups at 1 week, 1 month, and 2 months after injection were calculated and compared with control group and baseline.

### 2.4. Statistical Analysis

All statistical analyses were performed using IBM SPSS for Windows version 20.0 (SPSS, Chicago, IL, USA). The Kolmogorov–Smirnov test was used to assess the assumption of normality. Normally distributed variables were expressed as mean ± standard deviation, and the continuous variables that do not have normal distribution were expressed as median (25–75 percentiles). Also, categorical variables were summarized as counts (percentages). Comparisons of normally distributed continuous variables between groups were performed using student's *t*-test, and for non-normally distributed continuous variables, Mann–Whitney *U*-test was used. Lastly, sequential changes were analyzed by repeated-measures ANOVA and Friedman two-way ANOVA for normally and non-normally distributed variables, respectively. A two-sided *p* value < 0.05 was considered as statistically significant. Pearson and Spearman correlation analyses were used to determine the relationship between normally and non-normally distributed variables, respectively.

## 3. Results

We examined a total of 17 eyes of 17 patients with RVO (10 men, 7 women; age 60 ± 12 years; CRVO in 9 eyes, BRVO in 8 eyes). Median BCVA of study eyes was 12.00 (1.50–26.50) ETDRS letters; median intraocular pressure was 15.00 (13.00–18.00) mmHg, mean CMT was 551.24 ± 144.64 *μ*m, and mean SFCT was 265.06 ± 38.48 *μ*m. Changes of these parameters after DEX implantation are shown in Table 1. A total of 17 eyes of 17 age-and sex-matched control subjects (7 men, 10 women; 59 ± 10 years) met the inclusion criteria and were included for analysis. In control group, median BCVA was 55.00 (55.00–55.00) ETDRS letters, median IOP was 15.00 (13.00–17.00) mmHg, mean CMT was 276.24 ± 17.09 *μ*m, and mean SFCT was 229.09 ± 38.51 *μ*m.

**Figure 3:**
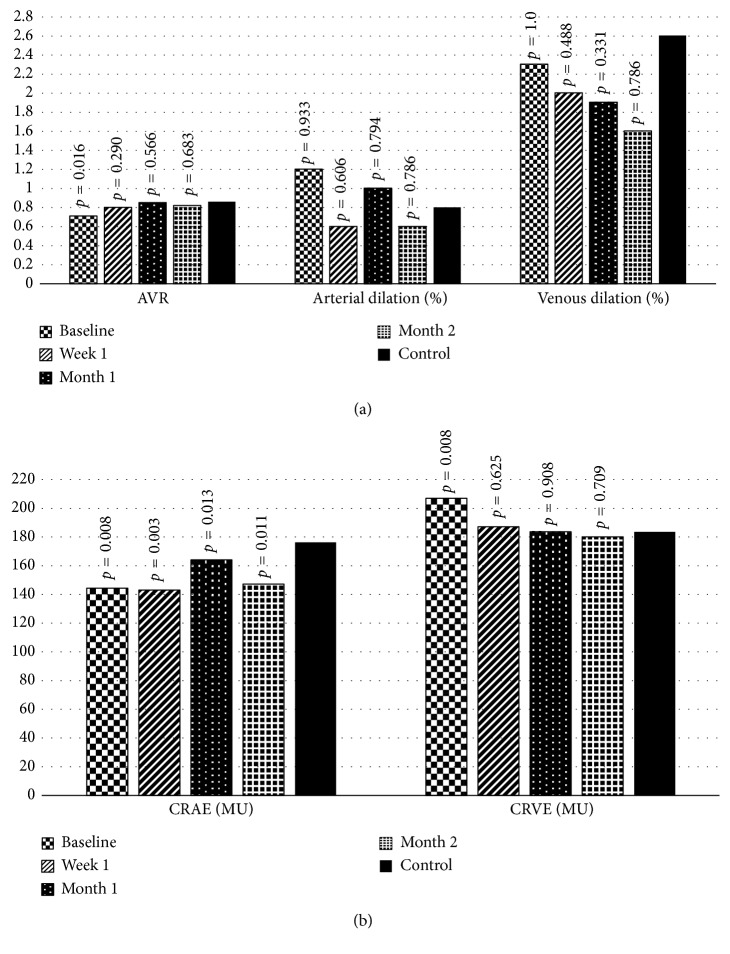
Comparisons of different static (a, b) and dynamic analysis (a) parameters in patients with macular edema secondary to retinal vein occlusion, at baseline (before injection) and 1 week, 1 month, and 2 months after injection. AVR = arteriovenous ratio; CRAE = central retinal artery equivalent; CRVE = central retinal vein equivalent; MU = measurement units; values are expressed as mean ± standard deviation and median (25–75 percentiles).

**Figure 4:**
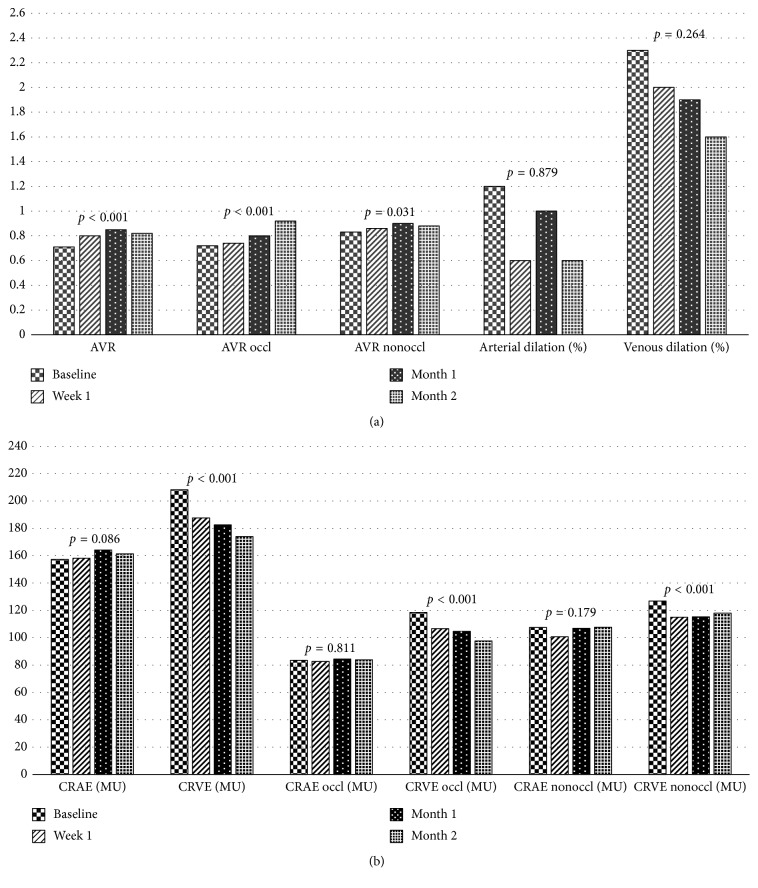
Comparisons of different static (a, b) and dynamic analysis (a) parameters in patients with macular edema secondary to retinal vein occlusion at baseline (before injection) and 1 week, 1 month, and 2 months after injection with control subjects. AVR = arteriovenous ratio; CRAE = central retinal artery equivalent; CRVE = central retinal vein equivalent; AVR nonoccl = arteriovenous ratio in nonoccluded quadrants of RVO and corresponding quadrants of controls; AVR occl = arteriovenous ratio in occluded quadrants of RVO and corresponding quadrants of controls; CRVE nonoccl = central retinal vein equivalent in nonoccluded quadrants of RVO and corresponding quadrants of controls; CRVE occl = central retinal vein equivalent in occluded quadrants of RVO and corresponding quadrants of controls; MU = measurement units; values are expressed as mean ± standard deviation and median (25–75 percentiles).

In static retinal vessel analysis, median AVR in control group was 0.86 (0.80–0.88)%. Baseline AVR in RVO group was statistically different from the control group (Figure 3(a)). Baseline AVR in RVO group was significantly lower than AVR at month 1 and month 2 (*p*=0.001 and *p* < 0.001, respectively) (Table 2 and Figure 4(a)). Considering 35 single occluded quadrants in 17 RVO eyes, AVR at week 1, month 1, and month 2 significantly increased compared to baseline (Table 2 and Figure 4(a)). Considering 24 nonoccluded quadrants (only BRVO), AVR significantly increased at month 1 and month 2 compared to baseline (Table 2 and Figure 4(a)).

Baseline CRVE in RVO group was statistically different from control group (Table 2 and Figure 4(b)). CRVE measurements for RVO group at week 1, month 1, and month 2 were significantly lower than baseline (*p* < 0.001, *p* < 0.001, and *p* < 0.001, respectively) (Table 2 and Figure 4(b)). But CRVE at week 1, month 1, and month 2 was different from control group (Figure 3(b)). CRVE in occluded quadrants significantly decreased at week 1, month 1, and month 2 compared to baseline (Table 2 and Figure 4(b)). CRVE in nonoccluded quadrants significantly decreased at week 1, month 1, and month 2 compared to baseline (Figure 4(b)).

CRAE in control group was 176.24 ± 22.45 MU. CRAE in RVO group was significantly lower at baseline, week 1, month 1, and month 2 compared to control group (Figure 3(b)). Baseline CRAE in RVO group was not statistically different from CRAE at week 1, month 1, and month 2 (Table 2 and Figure 4(b)). CRAE measurements for occluded quadrants did not change from baseline to month 2 (Table 2 and Figure 4(b)). Consecutive CRAE measurements for nonoccluded quadrants were not different from baseline (Table 2 and Figure 4(b)).

According to dynamic vessel analysis, median arterial and venous dilation rates during flicker stimulation in healthy control subjects were 0.80 (−0.05–1.80)% and 2.60 (1.70–3.25)%, respectively, and not different from baseline, week 1, month 1, and month 2 in RVO group (Figure 3(a)). Median arterial dilation rate was 1.20 (−0.95–3.15)%, and median venous dilation rate was 2.30 (0.90–5.00)% in RVO group.

In correlation analysis, we found positive correlation between BCVA and AVR at baseline (*r* = 0.686, *p*=0.002), week 1 (*r* = 0.755, *p*=0.001), month 1 (*r* = 0.697, *p*=0.002), and month 2 (*r* = 0.711, *p*=0.001). All the other correlations tested were not statistically significant.

## 4. Discussion

In this study, we investigated the impact of intravitreal DEX implant on retinal vessel functionality in consecutive measurements of eyes with ME secondary to RVO by using DVA. To improve our understanding of the vascular modification, we performed an analysis of vascular functionality using the DVA, which allows the noninvasive evaluation of retinal vessels both at steady state (static) and upon stimulation (dynamic). DVA measures retinal vessels response to flicker light easily, noninvasively, and accurately. Flicker-induced vasodilation of retinal vessels probably reflects endothelial function [23]. Flicker light stimulation of the retina has been used in healthy subjects to investigate the process of neurovascular coupling. With this mechanism, retina can regulate blood flow in response to neural activity. In fact, the increase of neural activity induced by flicker stimulation leads to retinal arterial and venous dilation because of the release of vasodilating factors, such as nitric oxide, from neural and endothelial cells [19]. The underlying role of nitric oxide in the neurovascular coupling was evidenced by studies [24, 25]. We found that AVR at month 1 and month 2 was higher than baseline, and CRVE at week 1, month 1, and month 2 was lower than baseline.

In CRVO, increase in venous outflow resistance is seen at the level of lamina cribrosa. But in BRVO, resistance is seen distally. Common adventitial sheath of artery and vein leads to more vulnerable vein [26]. In RVO, narrowing of arterial lumen caused by muscular hypertrophy of arterial wall secondary to hypertension and venous dilation distal to vein occlusion is seen. RVO triggers an inflammatory reaction of ocular cytokines [27]. Also, damage of vascular system and inflammatory cascade stimulates each other. Furthermore, dilation of vasculature and increased vascular permeability lead to leakage and edema within the retina [8, 28]. Intravitreal DEX may cause stabilization of endothelial cell tight junctions or downregulation of intraocular cytokine production in this pathologic pathway [10, 11]. In a study considering patients with ME secondary to RVO treated with DEX implant, a significant decrease in venous diameters was observed in the total study population and CRVO considering treatment, whereas none was observed in BRVO [29]. In our study, mean CRVE at baseline was significantly higher than control group because of dilation of veins secondary to occlusion in both CRVO and BRVO groups. But, with the effect of DEX, there was no significant difference between CRVE at week 1, month 1, and month 2 and control group. Corvi et al. [17] studied the effects of ranibizumab on vessel diameters by using DVA and revealed similarly that CRVE at baseline was significantly higher than control group. Also they experienced that CRVE at week 1, and month 1 were different than control group in contrast to our study. We believe that this was related to more potent effect of DEX.

In hypertensive patients, the initial response to elevated luminal pressure in is vasoconstriction which is evident by narrowing of retinal arteries and called as “vasoconstrictive phase.” [30] With time, elevated blood pressure causes endothelial damage and narrowing of vessels leading to the “sclerotic phase” [31]. CRAE in RVO group at baseline, week 1, month 1, and month 2 were significantly lower than control group. In [17], Corvi et al. found that CRAE in RVO group at baseline, week 1, and month 1 was not significantly different than control group. Causes of retinal vein occlusion were hypertensive retinopathy in our study population, so we experienced lower CRAE values.

In our study, while there was significant increase in AVR at month 1 and month 2 compared to baseline, there was no significant difference between week 1 and baseline. Because of venous dilation secondary to RVO, the artery-to-vein ratio was expected to be low at baseline. Depending on decrease in venous diameters with treatment, we experienced increase in AVR values. Corvi et al. [17] found that AVR in RVO group at week 1 and month 1 was not different than baseline. However, we believe that decrease in venous dilation should change AVR. In our study, there was significant difference between baseline AVR in RVO group and control group and this difference disappeared at week 1. This should be related with the decrease in venous dilation after 1 week. Corvi et al. [17] found that AVR in the RVO group at baseline, week 1, and month 1 was significantly lower than control group. It may be speculated that ranibizumab may be less effective in decreasing the venous dilation compared to DEX. For this reason, it may result with less increase in AVR. When we assess the only the 35 occluded quadrants of the patients (both CRVO and BRVO groups), we found a significant increase in baseline AVR values during follow-up. Similarly, consecutive measurements were not different from control group at month 1 and month 2.

The present study has limitations because of relatively low number of patients. Also, operative protocols of Dynamic Vessel Analyzer are not standardized because of it being new technology yet.

## 5. Conclusion

We used DVA to show the effects of DEX on vascular diameters in RVO patients. We experienced that retinal veins in patients with RVO were significantly larger compared to control group and intravitreal dexamethasone treatment reduced diameters of these veins. But further large-sample-sized studies are needed to confirm these results and vascular evaluation of RVO-affected retinal circulation.

## Figures and Tables

**Table 1:** Comparisons of different clinical parameters in patients with macular edema secondary to retinal vein occlusion, at baseline (before injection) and 1 week, 1 month, and 2 months after injection.

	Baseline	Week 1	Month 1	Month 2	*p*
BCVA	12.00 (1.50–26.50)^bc^	20.00 (5.00–27.50)	20.00 (8.50–36.00)^b^	20.00 (5.50–34.00)^c^	<0.001
CMT	551.24 ± 144.64^abc^	308.65 ± 71.38^a^	282.18 ± 77.05^b^	271.56 ± 54.19^c^	<0.001
SFCT	265.06 ± 38.48^c^	262.18 ± 38.17	260.82 ± 36.36	258.88 ± 39.55^c^	0.012
IOP	15.00 (13.00–18.00)^bc^	16.00 (13.00–9.00)	18.00 (14.00–19.50)^b^	19.00 (16.50–19.50)^c^	0.016

BCVA = best-corrected visual acuity; CMT = central macular thickness; SFCT = subfoveal choroidal thickness; IOP = intraocular pressure; values are expressed as mean ± standard deviation and median (25–75 percentiles). ^a^Comparison of baseline and 1 week (*p* < 0.05). ^b^Comparison of baseline and 1 month (*p* < 0.05). ^c^Comparison of baseline and 2 months (*p* < 0.05).

**Table 2:** Comparisons of different static and dynamic analysis parameters in patients with macular edema secondary to retinal vein occlusion, at baseline (before injection) and 1 week, 1 month, and 2 months after injection.

	Baseline	Week 1	Month 1	Month 2	*p*
AVR	0.71 (0.54–0.84)^bc^	0.80 (0.60–0.88)	0.85 (0.66–0.91)^b^	0.82 (0.70–0.95)^c^	<0.001
CRAE, MU	157.30 (115.00–180.10)	158.10 (108.15–173.05)	164.10 (115.25–173.95)	161.30 (119.85–179.50)	0.086
CRVE, MU	208.20 (192.40–224.30)^abc^	187.60 (174.10–204.35)^a^	182.50 (173.20–201.75)^b^	174.00 (158.40–199.85)^c^	<0.001
AVR occl	0.72 (0.54–0.82)^abc^	0.74 (0.55–0.99)^a^	0.80 (0.56–1.00)^b^	0.92 (0.69–1.01)^c^	<0.001
CRAE occl, MU	83.44 ± 20.20	82.64 ± 22.16	84.40 ± 22.88	83.79 ± 22.06	0.811
CRVE occl, MU	118.51 ± 26.96^abc^	106.62 ± 24.79^a^	104.60 ± 27.15^b^	97.62 ± 28.87^c^	<0.001
AVR nonoccl	0.83 (0.79–0.94)^bc^	0.86 (0.82–0.97)	0.90 (0.81–0.94)^b^	0.88 (0.82–0.99)^c^	0.031
CRAE nonoccl, MU	107.55 (86.52–112.75)	100.65 (86.40–112.37)	106.85 (84.05–114.22)	107.70 (83.82–112.95)	0.179
CRVE nonoccl, MU	126.88 ± 23.46^abc^	114.98 ± 29.20^a^	115.32 ± 23.36^b^	117.98 ± 22.62^c^	<0.001
Arterial dilation (%)	1.20 (−0.95–3.15)	0.60 (−0.20–1.60)	1.00 (0.00–2.50)	0.60 (-0.20–3.20)	0.879
Venous dilation (%)	2.30 (0.90–5.00)	2.00 (1.22–3.92)	1.90 (1.00–2.80)	1.60 (1.30–3.85)	0.264

AVR = arteriovenous ratio; AVR nonoccl = arteriovenous ratio in nonoccluded quadrants of RVO and corresponding quadrants of controls; AVR occl = arteriovenous ratio in occluded quadrants of RVO and corresponding quadrants of controls; CRAE = central retinal artery equivalent; CRAE nonoccl = central retinal artery equivalent in nonoccluded quadrants of RVO and corresponding quadrants of controls; CRAE occl = central retinal artery equivalent in occluded quadrants of RVO and corresponding quadrants of controls; CRVE = central retinal vein equivalent; CRVE nonoccl = central retinal vein equivalent in nonoccluded quadrants of RVO and corresponding quadrants of controls; CRVE occl = central retinal vein equivalent in occluded quadrants of RVO and corresponding quadrants of controls; MU = measurement units; values are expressed as mean ± standard deviation and median (25–75 percentiles). ^a^Comparison of baseline and 1 week (*p* < 0.05). ^b^Comparison of baseline and 1 month (*p* < 0.05). ^c^Comparison of baseline and 2 months (*p* < 0.05).

## Data Availability

The data used to support the findings of this study are available from the corresponding author upon request.
